# The PPARγ Locus Makes Long-Range Chromatin Interactions with Selected Tissue-Specific Gene Loci during Adipocyte Differentiation in a Protein Kinase A Dependent Manner

**DOI:** 10.1371/journal.pone.0086140

**Published:** 2014-01-20

**Authors:** Scott E. LeBlanc, Qiong Wu, A. Rasim Barutcu, Hengyi Xiao, Yasuyuki Ohkawa, Anthony N. Imbalzano

**Affiliations:** 1 Department of Cell and Developmental Biology, University of Massachusetts Medical School, Worcester, Massachusetts, United States of America; 2 Laboratory of Aging Research, State Key Laboratory of Biotherapy, West China Hospital, West China Medical School, Sichuan University, Chengdu, China; 3 Department of Advanced Medical Initiatives, JST-CREST, Faculty of Medicine, Kyushu University, Fukuoka, Japan; University of Maryland School of Medicine, United States of America

## Abstract

Differentiation signaling results in reprogramming of cellular gene expression that leads to morphological changes and functional specialization of a precursor cell. This global change in gene expression involves temporal regulation of differentiation-specific genes that are located throughout the genome, raising the idea that genome structure may also be re-organized during cell differentiation to facilitate regulated gene expression. Using in vitro adipocyte differentiation as a model, we explored whether gene organization within the nucleus is altered upon exposure of precursor cells to signaling molecules that induce adipogenesis. The peroxisome proliferator-activated receptor gamma (PPARγ) nuclear hormone receptor is a master determinant of adipogenesis and is required for adipose differentiation. We utilized the chromosome conformation capture (3C) assay to determine whether the position of the PPARγ locus relative to other adipogenic genes is changed during differentiation. We report that the PPARγ2 promoter is transiently positioned in proximity to the promoters of genes encoding adipokines and lipid droplet associated proteins at 6 hours post-differentiation, a time that precedes expression of any of these genes. In contrast, the PPARγ2 promoter was not in proximity to the EF1α promoter, which drives expression of a constitutively active, housekeeping gene that encodes a translation elongation factor, nor was the PPARγ2 promoter in proximity to the promoter driving the expression of the C/EBPα regulatory protein. The formation of the long-range, intergenic interactions involving the PPARγ2 promoter required the regulatory factor C/EBPβ, elevated cyclic AMP (cAMP) levels, and protein kinase A (PKA) signaling. We conclude that genome organization is dynamically remodeled in response to adipogenic signaling, and we speculate that these transient inter-genic interactions may be formed for the purposes of selecting some of the transcriptionally silent tissue-specific loci for subsequent transcriptional activation.

## Introduction

Maturation of a pre-adipocyte into a mature adipocyte involves significant changes in cellular structure and organization. Major changes in the expression of cytoskeletal structural proteins occur during adipogenesis of both cultured and primary cells; these changes have been linked to the notable transformation in cell morphology that occurs when preadipocytes differentiate into adipocytes [1]–[3]. Recent studies also report structural alterations that occur in the nuclear lamina and in the connections between the nucleus and cytoplasmic filaments as a function of adipogenic differentiation [4]. Furthermore, expression of the lamin associated polypeptide 2α (LAP2α) or reduction in the expression of lamins or of the lamin associated protein emerin modulates adipocyte differentiation [5]–[7], while mutations in nuclear lamina proteins have been associated with lipodystrophy syndromes [8], [9].

Other reports document organizational changes within the nucleus. These include reports of differentiation-dependent changes in chromosome territories [10], relocalization of adipogenic gene positioning within the nucleus relative to the nuclear lamina during differentiation [11], [12], and preferential association with SC-35 domains [13], which are nuclear structures that are enriched in factors involved in pre-mRNA metabolism and that may act to spatially link gene expression and mRNA processing [14], [15].

The pivotal controller of adipogenesis is PPARγ2, which is both necessary and sufficient for adipogenic differentiation [16]–[18]. While PPARγ2 expression does not occur until an intermediate stage in the differentiation program [19], it is well-established that the PPARγ2 promoter is marked by early binding of C/EBPβ and early changes in histone modification patterns and DNase I hypersensitivity [20]–[23] well in advance of PPARγ2 expression. This suggests the possibility that the early modulation of factor binding and chromatin structure at the PPARγ2 promoter may serve as a focal point for differentiation-dependent changes in higher order chromatin structure. Given these considerations, we wondered whether there was any spatial relationship between the PPARγ2 locus and the loci encoding other genes expressed during adipogenesis.

Most adipocyte-specific gene expression involves regulation by PPARγ2 [24]. PPARγ2 target genes are diverse and include genes encoding adipokines, which are hormones secreted by adipose that contribute to the regulation of energy balance of the organism and guide a number of physiological and pathological processes [25], [26]. Classic adipokines include adiponectin and leptin. Adiponectin levels are directly correlated with insulin sensitivity. Leptin is an appetite-sensitizing hormone for providing feedback for satiety, and both adipokines are believed to have high therapeutic potential [27], [28]. Another class of PPARγ2 target genes mediates lipid accumulation and storage. Lipid droplets are coated in proteins called perilipins, including perilipin 1 and adipose differentiation-related protein (ADRP), also called perilipin 2, which serve to protect the lipids contained within from adipose lipases [29], [30].

In this study, we document interactions between the PPARγ2 promoter and the promoters controlling the adipocyte hormone genes and perilipins 1 and 2 in differentiating 3T3-L1 adipocytes and in differentiating C3H10T1/2 mesenchymal cells using the chromosome conformation capture (3C) assay. Interestingly, the observed interactions between the PPARγ2 promoter and adipokine and perilipin promoters were most frequent at 6 hr following the addition of the adipogenic cocktail and decreased significantly at later time points. Thus the formation of these inter-genic interactions involving the PPARγ2 promoter preceded PPARγ2 gene expression and PPARγ2 target gene expression. Interactions were dependent upon the C/EBPβ transcriptional regulator. Additional experiments revealed that cAMP signaling was required for the formation of these long-range, inter-genic interactions, and that these interactions were also dependent on elevated cAMP levels and on the presence of protein kinase A.

## Materials and Methods

### Cell Culture and Retroviral Infections

3T3-L1 pre-adipocyte cells and C3H10T1/2 mesenchymal cells were obtained from ATCC and cultured in Dulbecco’s modified Eagle’s medium (DMEM) containing 10% calf serum or 10% fetal calf serum (FCS). For adipogenic differentiation, 2 day post-confluent cells were differentiated with a standard adipogenic cocktail (1 µg/ml insulin, 0.25 µg/ml dexamethasone, 0.5 mM isobutylmethylxanthine (IBMX) with 10% FCS) or with the indicated subset of components. Where indicated, forskolin (10 µM, Calbiochem) was added to serum-containing differentiation media lacking the differentiation cocktail components for the first two days of the differentiation process. 3T3-L1 cells were also pre-treated for 1 hour prior to administration of differentiation cocktail with either 10 µM H89 (Sigma) or 10 µM myr-PKI (Calbiochem). BOSC23 retroviral packaging cells were cultured in DMEM containing 10% FCS and were cycled through selective media every 1–2 months as described [31]. pSuperior-retro-puro (OligoEngine, Seattle, WA), pSuperior-retro-puro-PKAC1α, pSuperior-retro-puro-PKAC1β, and pSuperior-retro-puro-C/EBPβ [23] viral packaging was achieved by transfection of the plasmid into BOSC23 cells using Fugene6 (Roche) as described [23]. Collection of viral supernatant and infection of cells was also described previously [23].

### Chromatin Conformation Capture (3C) Assay

The 3C protocol was adapted from published methods [32]–[34]. Cells were cross-linked with 1% formaldehyde for 10 minutes at RT and quenched with 0.125M glycine for 5 min. Samples were harvested in PBS containing protease inhibitor cocktail (Sigma). Pellets were lysed in lysis buffer (10 mM Tris HCI pH 8.0, 10 mM NaCl, 0.5% Nonidet P-40) containing protease inhibitors, incubated on ice for 15 min, and dounced 10 times using pestle B, followed by another 15 minute incubation on ice. After removal of supernatant, nuclei pellets were re-suspended in restriction endonuclease buffer 2 (NEB; 50 mM NaCl,10 mM Tris-HCl,10 mM MgCl_2_,1 mM Dithiothreitol) and washed once in NEB buffer 2. SDS was added to a final concentration of 0.3%, followed by a 60 min incubation at 65 degrees C, followed by SDS sequestration using 1.8% Triton X-100 for 60 minutes at 37 degrees C. Samples were incubated with 350 units each of restriction enzymes StuI and PvuII (NEB) and were incubated overnight at 37 degrees C. After SDS-mediated inactivation of the enzymes for 30 minutes at 65°C (1.6% final concentration) and SDS-sequestration with 1% Triton X-100 at 37 degrees C for 60 min, 2X Takara Mighty Mix was added to each sample and incubated for 1 hr at 16 degrees C. Ligated samples were treated overnight with proteinase K (20 mg/mL) and RNase A (10 mg/mL), and DNA fragments were purified with a DNeasy Blood and Tissue kit (Qiagen). Samples were analyzed by realtime PCR using the Promega GoTaq Master Mix on a DNA Opticon (MJ Research) or an ABI StepOne Plus (Applied Biosystems) and interaction frequencies were normalized to the values of intra-genic interactions occurring at the endogenous TFIIH (ERCC3) locus [35] or a gene desert region located on chromosome 3 [36].

### Primer Sequences for 3C Assays

PPARγ2 promoter StuI site 1 (−132 bp from TSS) forward: 5′-GTAATGTACCAAGTCTTGCCAAAGCAGCAG

Leptin PvuII Forward: 5′-TCACAGGATGAAATGAGACGACTGTTCTTCG

Adiponectin PvuII Forward: 5′-TAGAGAATGGCCAAAGCCTGGAAACAGGA

Perilipin-1 PvuII Forward: 5′-TGTTTCTGAGGAGGAGACCTT

Perilipin-2 PvuII Forward: 5′-AATGAGTGGCCCCAGAGTC

C/EBPα StuI Forward: 5′- CTAGGTTGCTGGTCCAAAGCAGTCTCCAAC

EF1α StuI Forward: 5′-GTCGCCTTGGACGTTCTTTT

ERCC3 −0.9 Forward: 5′-TGTAGTTCTGTGGCTAGAACCTG

ERCC3 3.7 Forward: 5′-TGATGCAGAAGGATGCTGAC

Chromosome 3 Gene desert region:

GDR StuI Forward 5′-CCATTTAGAATCGTGGCAGGTCTAT

GDR PvuII Forward 5′-ATACATTGAAATGAGTCCTCCACAT

### Protein Extracts and Western Blots

Whole cell extracts were prepared and quantified and used for western blotting as described [37]. Primary antibodies were PKAC1α (Santa Cruz; sc-903), PKAC1β (Santa Cruz: sc-904), C/EBPβ (Santa Cruz: sc-150), and p85 phosphatidylinositol-3-kinase (Millipore: 06–195).

### Statistics

Results are indicated as the average of three independent experiments +/− standard deviation. Data were statistically analyzed by a one-tailed t-test.

## Results

### The PPARγ2 Promoter Makes Long-range Interactions with Adipokine and Perilipin Promoters in Differentiating Adipocytes

Numerous molecular changes occur at the PPARγ2 promoter within hours following the onset of adipogenic differentiation signaling. Restriction endonuclease accessibility is increased within 30 minutes, c-fos binds within 1 hour, C/EBPβ binds within 2 hours, and DNAse I hypersensitivity was reported by 2 hours [21], [23]. All of these events occur well before the onset of PPARγ2 mRNA synthesis [38].

We hypothesized that the onset of differentiation signaling might cause rearrangement of genomic chromatin structure. Because of the requirement for and central role of the PPARγ2 protein in the adipogenic differentiation process, we investigated whether the PPARγ locus might be a region where changes in genomic organization occur. We utilized 3C methodology [32] to assess relative changes in gene organization. The 3C technique utilizes cross-linking to identify regions of chromatin that are in close juxtaposition to each other and has allowed identification of inter-chromosomal interactions as well as long-range interactions between non-contiguous sequences on the same chromosome [39]–[41].

**Figure 1:**
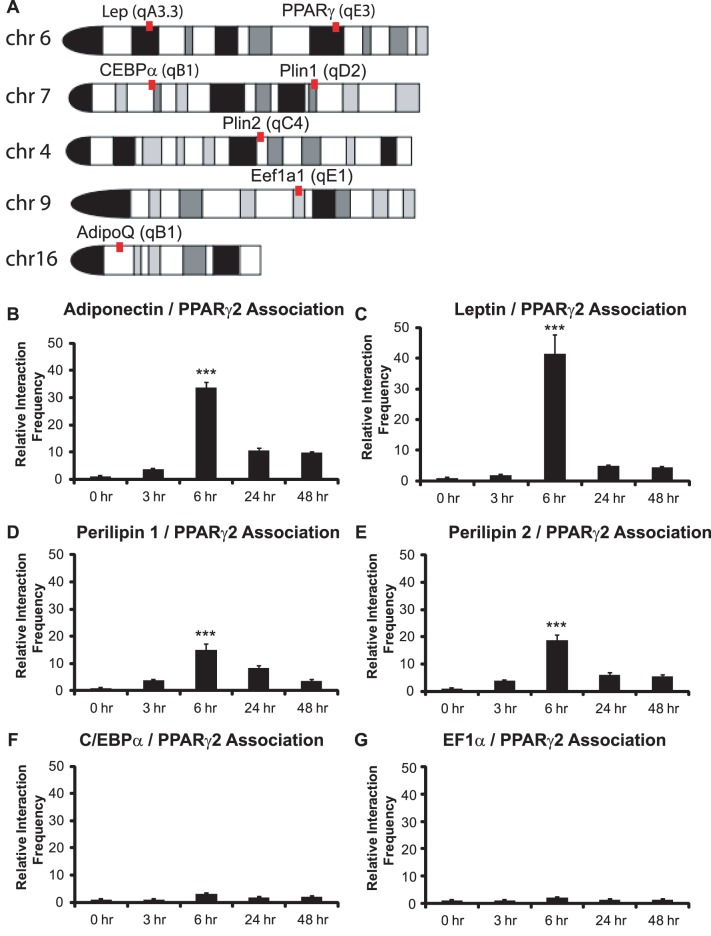
The PPARγ2 promoter maximally interacts with adipokine and perilipin gene promoters in 3T3-L1 cells 6 hours post-differentiation. (A) Schematic map of chromosomal locations of loci of interest. Lep: leptin; Plin1: perilipin 1; Plin2: perilipin 2; Eef1α1: EF1α; AdipoQ; adiponectin. 3C assays were performed at the indicated times to measure long-range, intergenic interactions between the PPARγ2 promoter and (B) the adiponectin promoter, (C) the leptin promoter, (D) the perilipin 1 promoter, (E) the perilipin 2/ADRP promoter, (F) the C/EBPα promoter, or (G) the EF1α promoter. Interaction frequencies are presented relative to the intragenic interaction frequency observed between different regions of the TFIIH locus, which served as a normalization control. The interaction frequency at 0 h was set to 1; the interaction frequencies for the remaining time points are presented relative to that value. Results indicate the average of three independent experiments +/− standard deviation. Values for the timepoint of maximum interaction frequency (6 h) were compared to values at time 0 by one-tailed t-test. ***p<0.001.

For initial 3C experiments, we sought to determine whether the PPARγ locus is in close physical proximity to other adipogenic loci in undifferentiated pre-adipocytes or in pre-adipocytes that have been exposed to differentiation signaling. We designed primers to the PPARγ2 promoter and to promoters from four known PPARγ2 target genes, those controlling expression of the adipokines, leptin and adiponectin, and those regulating the expression of two genes involved in lipid metabolism and storage, perilipin 1 and perilipin 2/ADRP. We also decided to test for interactions between the PPARγ2 promoter and the C/EBPα promoter, which is not bound by PPARγ2 but is regulated by PPARγ2 protein via displacement of co-repressor proteins [42]. Finally we examined interactions between the PPARγ2 promoter and the promoter of the EF1α gene, which is a constitutively active housekeeping gene encoding the eukaryotic translation initiation factor 1 alpha 1. The 3C assay is limited by the choice of restriction sites within range of the promoter, so we took advantage of a StuI restriction site within 150 bp of the transcription start site (TSS) of PPARγ2 and thus could assess interactions between genomic sites cut with Stu I and with PvuII, another blunt end cutter. The PPARγ locus is in band 6qE3 whereas the leptin locus is situated at 6qA3.3 on chromosome 6, 85 Mbp away. The adiponectin locus is located on chromosome 16, while the perilipin 1 and perilipin 2/ADRP loci are located at chromosomes 7 (7qD2) and 4, respectively. The C/EBPα and the EF1α loci are located on chromosomes 7 (7qB1) and 9, respectively (Fig. 1A). Thus any interactions observed would be long-range within the same chromosome or would be inter-chromosomal.

Initial experiments were performed as a time course using 2-day confluent 3T3-L1 preadipocytes (time 0) and cells treated with the standard adipogenic differentiation cocktail of insulin, dexamethasone, and IBMX for the indicated number of hours. Interaction frequencies were normalized to the interaction frequencies observed in each sample for TFIIH locus interactions, and were presented as relative values with the frequency observed at time 0 set to 1. The results indicate a spike in interaction frequency between the PPARγ2 promoter and the adiponectin promoter at 6 hours post-differentiation, which precedes PPARγ2 gene expression, with interaction frequencies at 24 and 48 hours greatly reduced in comparison to the 6 hour timepoint (Fig. 1B). Nearly identical results were observed for the interaction frequency between the PPARγ2 promoter and the leptin promoter (Fig. 1C). Interaction frequencies between the PPARγ2 promoter and the perilipin 1 and perilipin 2/ADRP promoters showed a similar result (Fig. 1D–E), though the relative interaction frequencies for these promoters was reduced by ∼40–60%. In contrast, interactions between the PPARγ2 and C/EBPα promoters were minimal (Fig. 1F). Interactions between the PPARγ2 promoter and the constitutively active EF1α promoter were also at background level (Fig. 1G). The temporal nature of the interactions between the PPARγ2 promoter and the two adipokine and the two lipid droplet associated protein gene promoters, along with the lack of interactions between the PPARγ2 promoter and the C/EBPα or EF1α promoters, indicates a level of specificity for the observed long-range genomic interactions.

The data in Figure 1 and in all subsequent figures were normalized to interaction frequencies detected by 3C at the TFIIH locus. The TFIIH locus was chosen to control for interactions because it exhibits a specific three-dimensional structure that appears to be invariant between different cell types and under different conditions. Thus the interaction frequency between sequences from different parts of the TFIIH locus are expected to be equivalent in all samples and can be used to control for sample to sample variation, as previously demonstrated [35]. As an additional control, we normalized the data in Figure 1 to the interaction frequency determined by 3C analysis of a gene desert region on chromosome 3, as done by others [36]. The data obtained by normalization to the interaction frequencies detected at the gene desert region is indistinguishable from the data normalized to TFIIH locus interactions (Fig. S1).

**Figure 2:**
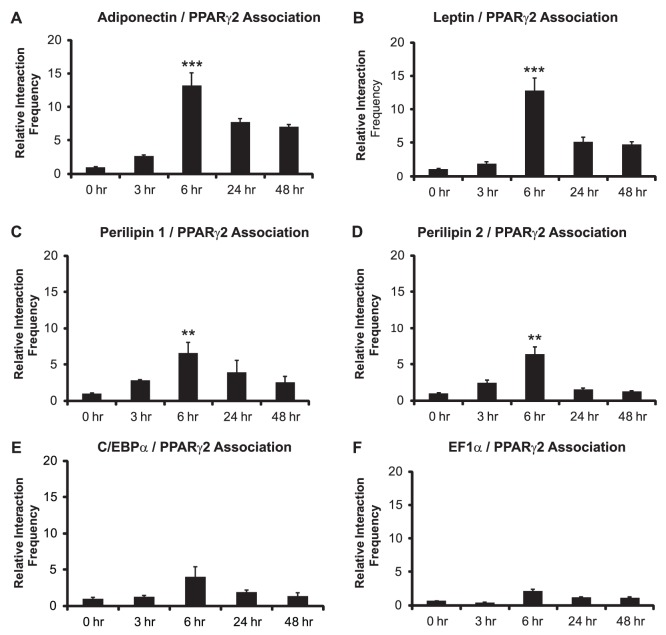
The PPARγ2 promoter maximally interacts with adipokine and perilipin gene promoters in C3H10T1/2 cells 6 hours post-differentiation. 3C assays were performed at the indicated times to measure long-range, intergenic interactions between the PPARγ2 promoter and (A) the adiponectin promoter, (B) the leptin promoter, (C) the perilipin 1 promoter, (D) the perilipin 2/ADRP promoter, (E) the C/EBPα promoter, or (F) the EF1α promoter. Interaction frequencies are presented relative to the intragenic interaction frequency observed between different regions of the TFIIH locus, which served as a normalization control. The interaction frequency at 0 h was set to 1; the interaction frequencies for the remaining time points are presented relative to that value. Results indicate the average of three independent experiments +/− standard deviation. Values for the timepoint of maximum interaction frequency (6 h) were compared to values at time 0 by one-tailed t-test. ***p<0.001; **p<0.01.

To confirm the results, we utilized a different cellular model for adipocyte differentiation. C3H10T1/2 cells are mesenchymal precursor cells that are capable of differentiating along the adipocyte lineage in response to the same cocktail used to induce 3T3-L1 cell differentiation [43]. The promoters for the adipokines as well as the perilipin genes associated with the PPARγ2 promoter maximally at the 6 hr timepoint (Fig. 2A–D), while interactions with the C/EBPα and EF1α promoters were minimal (Fig. 2E–F). The relative interaction frequencies between interacting promoters in the differentiating C3H10T1/2 cells were lower than those observed in the 3T3-L1 cells. The reason(s) for these differences is not known, though it is unrelated to the efficiency of differentiation, as Oil Red O staining of plates of both cell types maintained in differentiation conditions for 4 days showed equivalent levels of Oil Red O stained cells (data not shown).

### Long-range Inter-genic Interactions Involving the PPARγ2 Promoter Require C/EBPβ

**Figure 3:**
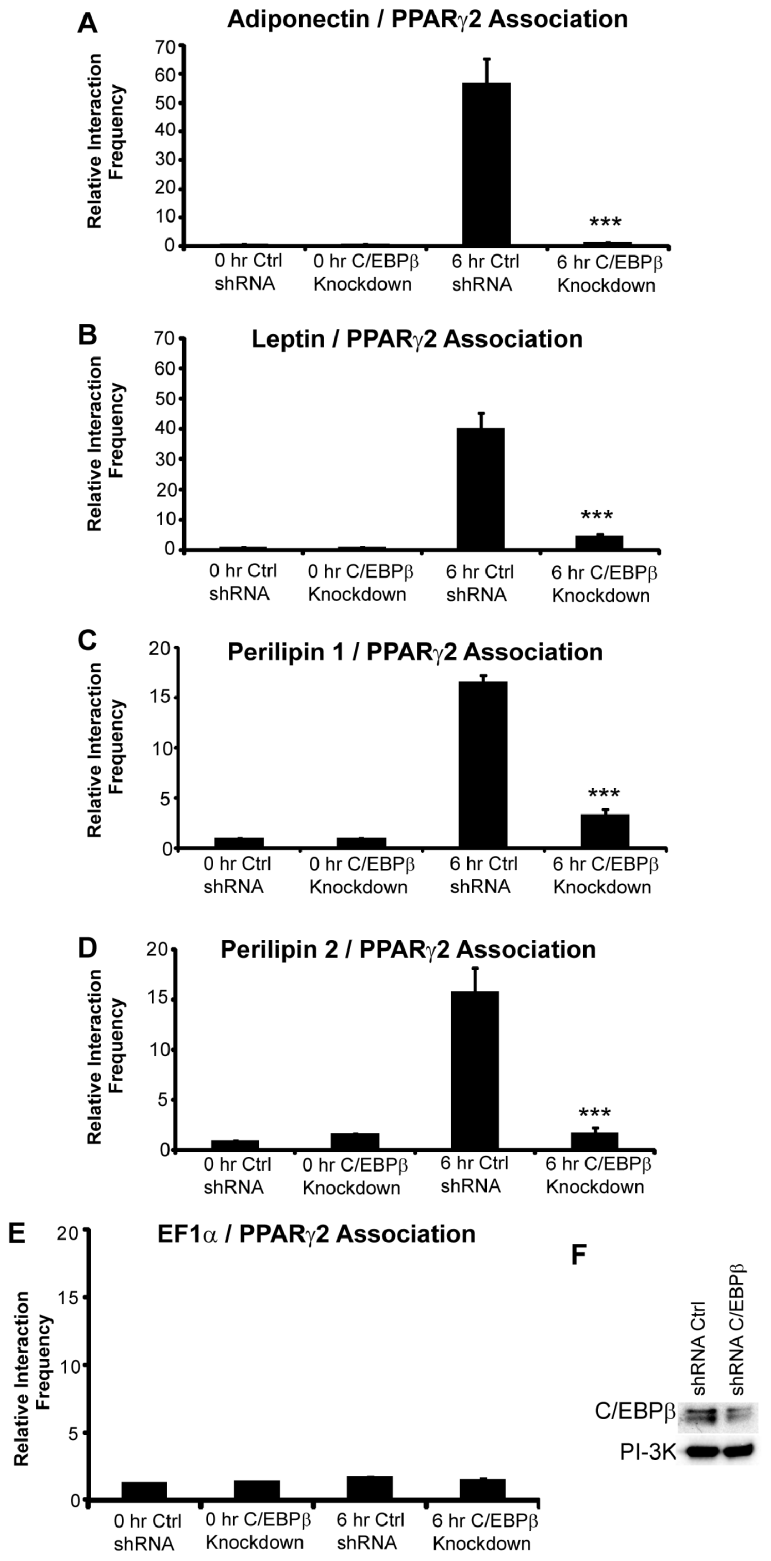
C/EBPβ is required for the formation of long-range, inter-genic interactions between the PPARγ2 promoter and the indicated promoters. Cells exposed to control shRNA or shRNA targeting C/EBPβ were assayed at 0 h and 6 h post-differentiation. Interaction frequencies between the PPARγ2 promoter and (A) the adiponectin promoter, (B) the leptin promoter, (C) the perilipin 1 promoter, (D) the perilipin 2/ADRP promoter, or (E) the EF1α promoter are presented relative to the intragenic interaction frequency observed between different regions of the TFIIH locus, which served as a normalization control. The interaction frequency at 0 h in the control shRNA treated cells was set to 1; the interaction frequencies for the other samples are presented relative to that value. Results indicate the average of three independent experiments +/− standard deviation. Values for the 6 h control shRNA treated samples were compared to values for the samples treated with C/EBPβ shRNA by one-tailed t-test. ***p<0.001. (F) Western blots indicating C/EBPβ protein levels in control and C/EBPβ shRNA treated cells. PI-3 kinase levels were monitored as a loading control.

C/EBPβ is a critical regulator of adipogenesis, factoring into the activation of both C/EBPα and PPARγ2 gene expression, which are essential for adipogenic differentiation [44], [45]. Since C/EBPβ binds to the PPARγ2 promoter within the first 2 hours of differentiation and is required to maintain open chromatin structure at the PPARγ2 promoter [23], [46], we asked whether C/EBPβ was similarly required for the long-range chromatin interactions involving the PPARγ2 locus at 6 h post-differentiation. 3T3-L1 cells were infected with retrovirus expressing a control shRNA or shRNA previously shown to reduce C/EBPβ levels [23] and subsequently differentiated for 6 h. As indicated in Fig. 3A–E, the interaction frequency for PPARγ2 with each of its interacting loci was almost completely eliminated. Control western blots demonstrated that C/EBPβ protein levels were reduced in cells expressing the shRNA against C/EBPβ (Fig. 3F). These results suggest that C/EBPβ and the initial events that occur in the first few hours of PPARγ2-driven gene activation during adipogenesis are required for subsequent formation of long-range chromatin interactions involving the PPARγ2 locus.

### Long-range Inter-genic Interactions Involving the PPARγ2 Promoter Require PKA Signaling

**Figure 4:**
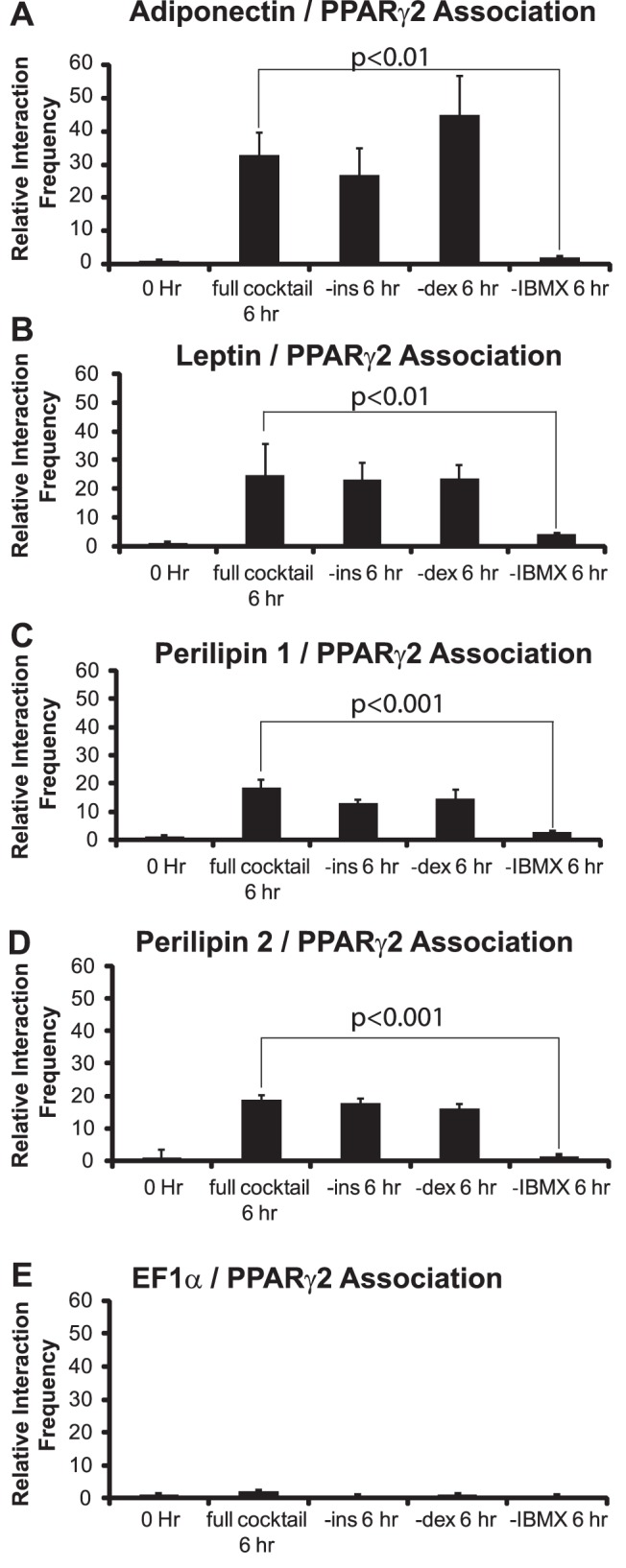
Elevation of cAMP levels is required for the formation of long-range, inter-genic interactions between the PPARγ2 promoter and the indicated promoters. The EF1α promoter is shown as a control. 3C assays were performed on 3T3-L1 cells at 0 or at 6 h post-differentiation in the presence of complete differentiation cocktail or in cocktail lacking the indicated component. Interaction frequencies were quantified as described in the legends for Figures 1–2. Results indicate the average of three independent experiments +/− standard deviation. Values for the 6 h samples differentiated in complete cocktail were compared to values for the samples differentiated in cocktail lacking IBMX by one-tailed t-test. *p* values are indicated on the graph. ins; insulin. dex; dexamethasone.

The cocktail used to induce differentiation contains activators of different signaling pathways. These include dexamethasone, a synthetic glucocorticoid agonist, IBMX, a phosphodiesterase inhibitor that elevates cellular cAMP levels, and insulin, a well-known modulator of glucose uptake. To determine whether one or more of these signaling molecules were required for the formation of interactions between the PPARγ2 promoter and the other promoters, we performed a series of dropout experiments where a series of differentiation cocktails, each lacking one component, were used to stimulate differentiation. Cocktails lacking dexamethasone or insulin had no effect on the interaction frequencies observed between the PPARγ2 promoter and the adipokine and the perilipin promoters 6 hours post-differentiation (Fig. 4). As expected under these conditions, adipogenic differentiation was inhibited (data not shown). However, removal of IBMX blocked the formation of the long-range inter- and intra-genic interactions at this time point (Fig. 4), along with differentiation into mature adipocytes.

**Figure 5:**
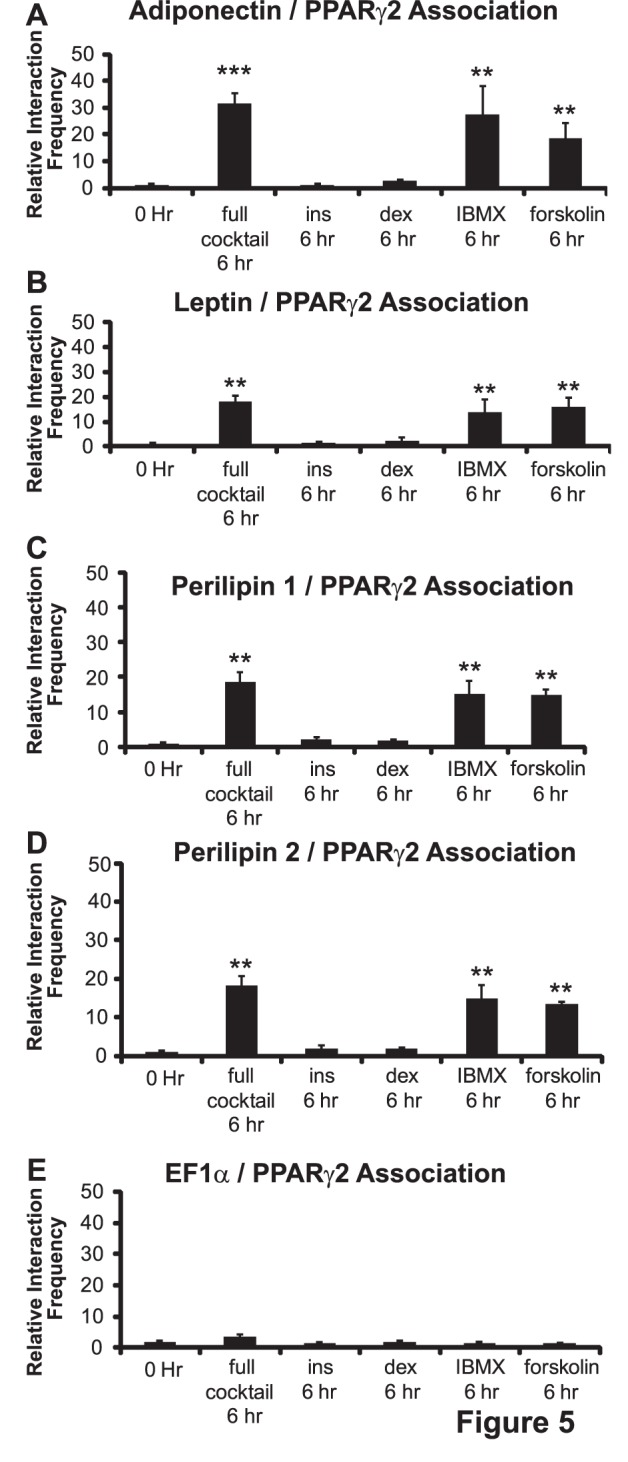
Elevation of cAMP levels is sufficient for the formation of long-range, inter-genic interactions between the PPARγ2 promoter and promoters. The EF1α promoter is shown as a control. 3C assays were performed on 3T3-L1 cells at 0 or at 6 h post-differentiation in the presence of complete differentiation cocktail or in serum-containing media containing only the indicated component. Interaction frequencies were quantified as described in the legends for Figures 1–2. Results indicate the average of three independent experiments +/− standard deviation. Values for the 6 h samples differentiated in complete cocktail were compared to values for the samples differentiated for 6 h in cocktail containing serum and IBMX or serum and forskolin by one-tailed t-test. ***p<0.001; **p<0.01. ins; insulin. dex; dexamethasone.

Since IBMX was necessary for the formation of the interactions between PPARγ2 and the other gene promoters, we next decided to test whether IBMX was sufficient to form the interactions. 3C assays were performed on 3T3-L1 cells following 6 hours of exposure to the complete cocktail or to each of the signaling components alone. Neither insulin alone nor dexamethasone alone supported the formation of the observed long-range interactions whereas exposure to IBMX alone did support the formation of the interactions between the PPARγ2 and the adipokine and perilipin promoters (Fig. 5). Since IBMX elevates cAMP levels, we sought to confirm that cAMP elevation was sufficient to promote intergenic interactions. Exposure of 3T3-L1 cells to serum-containing media containing forskolin, an activator of adenylyl cyclase [47], but lacking the other differentiation cocktail components promoted interactions between PPARγ2 and the adipokine and perilipin promoters 6 hours later (Fig. 5). Collectively, these findings indicate that that enhanced cAMP levels are critical for the formation of the long-range interactions between PPARγ2 and other adipogenic genes.

**Figure 6:**
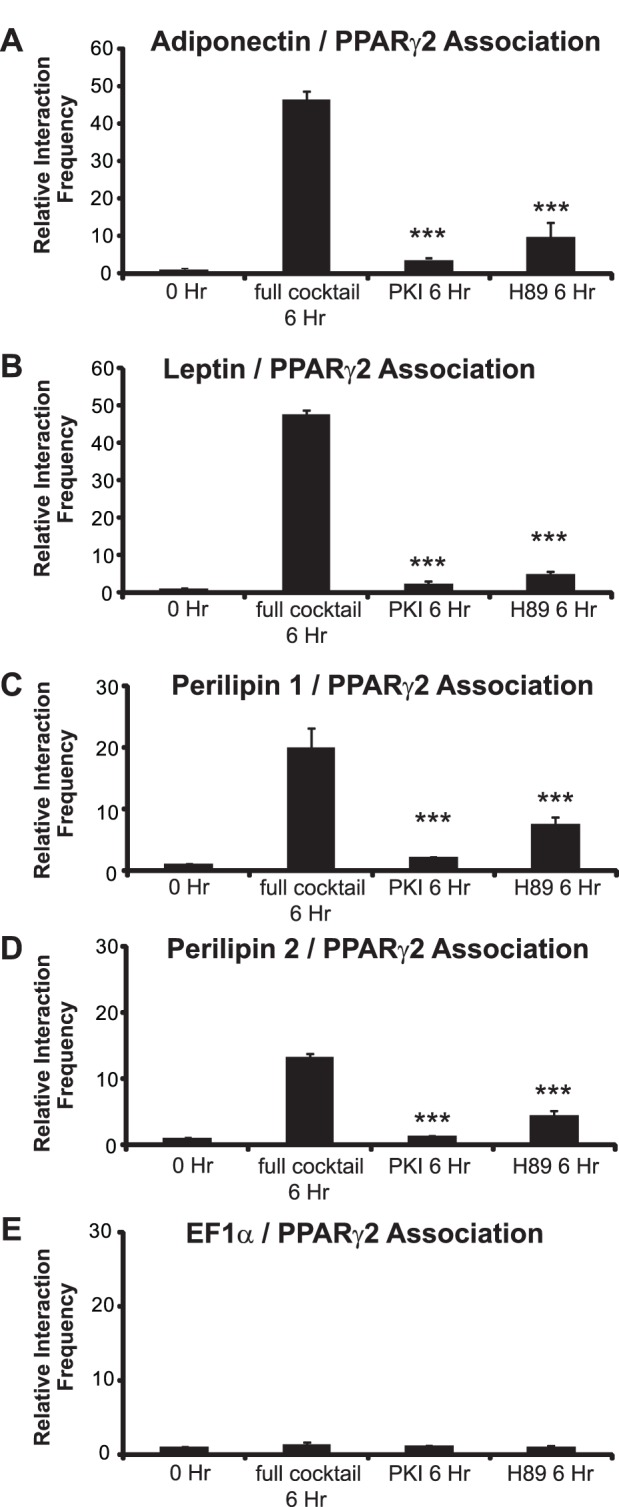
PKA inhibitors prevent the formation of long-range, inter-genic interactions between the PPARγ2 promoter and (A) the adiponectin promoter, (B) the leptin promoter, (C) the perilipin 1 promoter, (D) the perilipin 2/ADRP promoter. The EF1α promoter (E) is shown as a control. Interaction frequencies were quantified as described in the legends for Figures 1–2. Results indicate the average of three independent experiments +/− standard deviation. Values for the 6 h samples differentiated in complete cocktail were compared to values for the samples differentiated for 6 h in complete cocktail containing the PKI or H89 inhibitors by one-tailed t-test. ***p<0.001.

The cAMP-dependent protein kinase, also known as protein kinase A (PKA), is activated by elevated levels of cAMP. PKA consists of a tetrameric holoenzyme [48] that contains two catalytic subunits, Cα and Cβ [49]. To assess the requirement for PKA in the formation of the long-range genomic interactions involving the PPARγ2 promoter, we inhibited PKA activity by treating cells with PKI, a competitive, synthetic peptide inhibitor of PKA derived from the active domain of the naturally occurring PKA inhibitory protein also called PKI [50], or with H89, an isoquinolinesulfonamide selected for potent and specific PKA inhibition [51]. PKA inhibition by either compound inhibited long-range interactions between PPARγ2 promoter and the perilipin, leptin, and adiponectin promoters (Fig. 6). These experiments demonstrate the requirement for PKA in mediating long-range chromatin interactions involving the PPARγ2 promoter.

**Figure 7:**
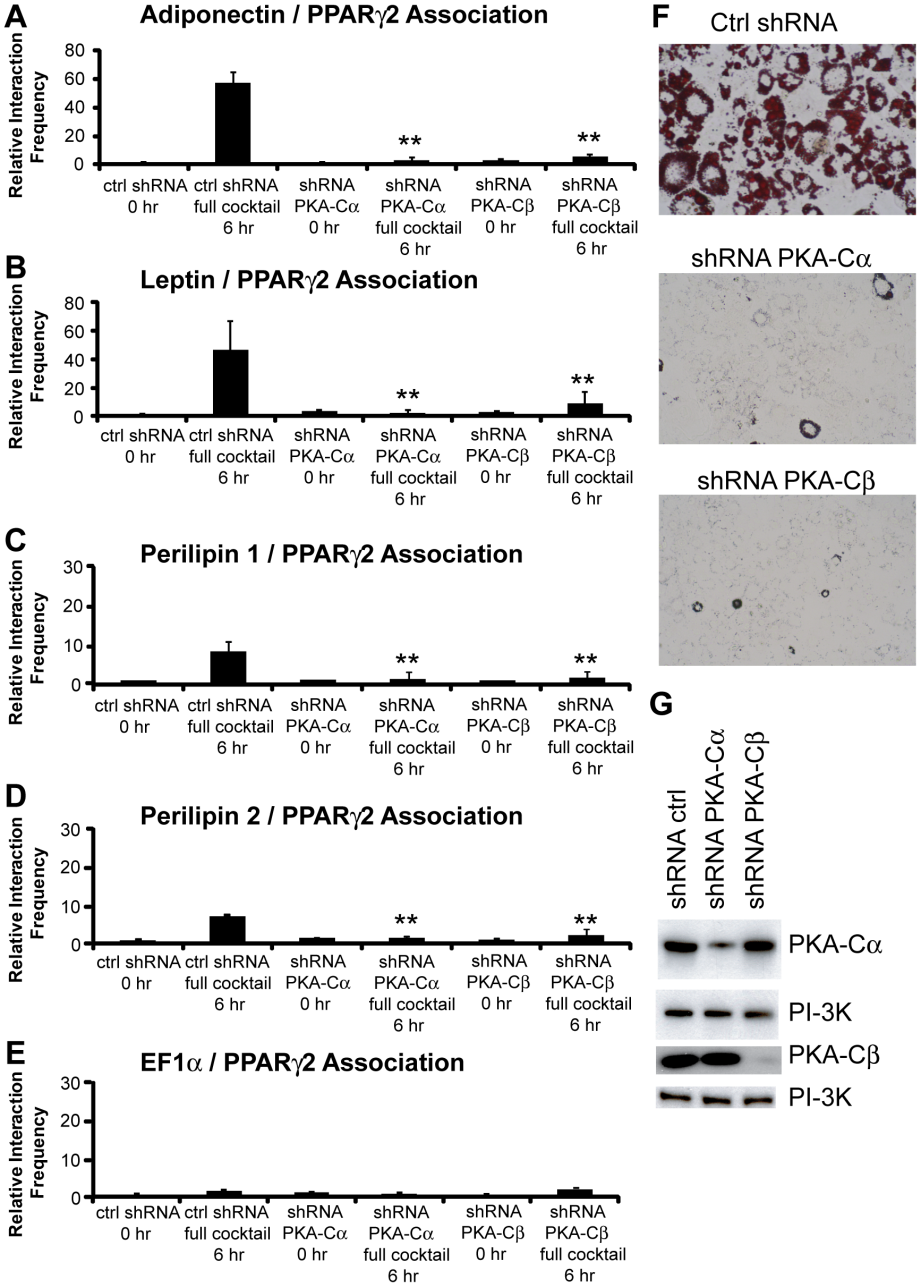
PKA is required for the formation of long-range, inter-genic interactions between the PPARγ2 promoter and (A) the adiponectin promoter, (B) the leptin promoter, (C) the perilipin 1 promoter, (D) the perilipin 2/ADRP promoter. The EF1α promoter (E) is shown as a control. 3T3-L1 cells infected with retroviral vectors expressing control shRNA or shRNA targeting the PKA-Cα or PKA-Cβ subunits were generated (see Methods). 3C assays were performed on these cells at 0 or at 6 h post-differentiation in the presence of complete differentiation cocktail. Interaction frequencies were quantified as described in the legends for Figures 1–2. Results indicate the average of three independent experiments +/− standard deviation. Values for the 6 h samples treated with control shRNA were compared to values for the 6 h samples treated with PKA-Cα or PKA-Cβ shRNA by one-tailed t-test. **p<0.01. (F) Oil Red O staining of infected 3T3-L1 cells allowed to differentiate for 4 days. (G) Western blots indicating the levels of the PKA-Cα or PKA-Cβ subunits in the infected cells. PI-3K levels were monitored as a loading control.

To directly determine whether PKA is involved in the formation of long-range interactions between the PPARγ2 and PPARγ2-target gene promoters, we used shRNAs against either the Cα or Cβ subunit to reduce PKA protein levels in 3T3-L1 pre-adipocytes. Previously we demonstrated that knockdown of either catalytic subunit blocked 3T3-L1 cell differentiation, in part because chromatin accessibility changes at the PPARγ2 promoter were inhibited [23]. Knockdown of either subunit inhibited inter-chromosomal interactions between the PPARγ2 promoter and the adiponectin and the perilipin promoters along with the intra-chromosomal interaction between the PPARγ2 promoter and the leptin promoter (Figure 7A–E). Oil Red O staining confirmed that knockdown of the PKA catalytic subunit inhibited differentiation and western blotting demonstrated that the PKA catalytic subunit protein levels were, in fact, reduced (Figure 7F–G). We conclude that PKA is required for PPARγ2 promoter involvement in long-range interactions with other promoters.

## Discussion

In this study, we provide evidence that long-range chromatin interactions occur between the promoter controlling the expression of the master regulator of adipogenesis, PPARγ2, and promoters controlling the expression of adipokines and lipid-droplet associated proteins in a transient manner that requires C/EBPβ and protein kinase A prior to the onset of the expression of these genes. While we do not understand the functional ramifications of such associations, we speculate that they may relate to early events in adipogenesis that prepare or mark tissue-specific loci for subsequent gene activation.

Cytological evidence supports the idea of chromosomal domains in interphase cells, however, the relative positions of chromosomal domains can vary by cell type and by the activation of signaling and/or gene induction pathways [52], [53]. The interfaces between chromosomal domains provide an environment in which inter-chromosomal interactions may form. Inter-chromosomal interactions have been documented previously. Such associations have often been correlated with the regulation of gene expression [54]–[61]. The transient nature of the observed long-range interactions suggests that if the interactions are part of the process through which gene activation is initiated, they could reflect the localization of the regulatory sequences with machinery that modifies the local chromatin structure. Obvious candidates for such machinery would be enzymes that post-translationally modify the promoter histones. Alternatively, or in addition, promoter sequences could have nucleosomal composition altered by addition or removal of histone variants, linker histones, or non-histone chromatin proteins. Recent work has suggested that deposition of the variant histone H3.3 marks skeletal muscle-specific genes for activation in myoblasts even before differentiation is initiated [62]. Precise time course experiments would be required to determine whether H3.3 deposition or the addition or removal of any histone or histone modification tracks with the formation of long-range interactions between the PPARγ2 and other promoters. This model hypothesizes that the enzymatic modifications occur at one or more specific sites in the nucleus. Determining whether such a site corresponds to a recognizable nuclear domain (*e.g.* – PML body, splicing speckle) or not would require immuno-FISH or similar approaches. Regardless, if fixed sites of enzymatic modification of genomic DNA exist, it would necessitate localization of the promoter sequences to that site. The transient nature of the interactions between the adipogenic gene promoters would then suggest that any chromatin modifications or structural changes that were made would be reasonably stable and not subjected to a dynamic cycle of addition/removal of the specific mark and/or histone. Whether the observed inter-genic associations relate to the formation or maintenance of transcription factories, where multiple genes are co-localized with pol II and active transcription [63]–[65], requires additional investigation.

The possibility of a link between the promoter interactions between PPARγ2 and the selected adipokine and perilipin genes and functions related to subsequent adipocyte-specific gene expression is further complicated by the selectivity of the interactions that were observed. The relative interaction frequencies observed between PPARγ2 and the C/EBPα promoter were minimal and equivalent to those observed between the PPARγ2 and EF1α promoters, which represent background levels. Clearly, the C/EBPα gene is activated during adipogenesis, yet unlike the other gene promoters examined, regulation of C/EBPα expression by PPARγ2 seems to be limited to post-transcriptional regulation in which the PPARγ2 protein functions to release HDAC1 from the C/EBPα promoter as part of the activation process [42]. Analyses of ChIP-seq data from 3T3-L1 cells and adipose tissue do not reveal significant binding of PPARγ2 or C/EBPα in the proximal promoter region of the C/EBPα gene [66], [67]. Perhaps different mechanisms occur for controlling the organization and activation of the C/EBPα gene during differentiation. Alternatively, perhaps C/EBPα participates in similar interactions with other adipogenic genes, but gene associations involving the C/EBPα promoter do not overlap with interactions involving the PPARγ2 locus. These possibilities remain to be tested; a more genome-wide approach would likely be more productive than the candidate gene approach taken here.

Regardless of the functional consequences of these long-range interactions, their existence raises the interesting question of how genomic DNA “moves” around the nucleus. The concept of rapid repositioning of genomic sequences is controversial. There are previous reports in the literature indicating that rapid, locus-specific genomic rearrangements occur [55], [68], [69]. Interestingly, the movement of the loci analyzed has been linked to the activity of nuclear actin and myosin as well as to dynein light chain [55], [68]. However, the reported rapid repositioning of estrogen-responsive genes upon estrogen treatment [55] has been questioned [70], and other work indicates that movement of chromosome territories is limited during interphase [71], [72]. Clearly there is a need for substantial additional work to better understand the dynamics of locus-specific chromatin organization and re-organization during physiological processes such as cell differentiation.

Figure 4, Figure 5, Figure 6, Figure 7 demonstrate that formation of the long-range interactions required elevated cAMP levels and the catalytic subunits of protein kinase A. Adipogenic signaling in both the 3T3-L1 and the C3H10T1/2 cell culture models requires addition of IBMX, which is a phosphodiesterase inhibitor that elevates cellular cAMP levels, which in turn activates PKA. During the early stages of adipogenic differentiation, PKA phosphorylates CREB, the cAMP regulatory element-binding protein, which contributes to the transcriptional activation of C/EBPβ [73], [74], at least in part by binding to the C/EBPβ promoter within an hour of the induction of differentiation [73]. At the PPARγ2 promoter, chromatin accessibility and c-fos binding occur within an hour of the induction of differentiation, and C/EBPβ binding occurs two hours post-differentiation. All of these events are dependent on elevation of cAMP levels and PKA function [23]. An additional intriguing finding is that PKA has the potential to form a complex with dynein [75], which as previously stated, was implicated as a molecular motor that is necessary for gene-specific organizational changes in estrogen treated cells [55]. Thus a potential relationship between PKA and a factor linked to genomic organization exists that might explain the observed formation of long-range intergenic interactions between selected adipogenic promoters. Though the connections between PKA, C/EBPβ, the molecular events at the PPARγ2 promoter, and the involvement of the PPARγ2 promoter in long-range intergenic interactions require further clarification, it seems clear that signaling through PKA is intricately involved in modulating the gene organization and transcriptional potential of tissue-specific gene expression during adipocyte differentiation.

## Supporting Information

Figure S1 **The PPARγ2 promoter maximally interacts with adipokine and perilipin gene promoters in 3T3-L1 cells 6 hours post-differentiation.** 3C interaction frequencies between PPARγ2 and the indicated promoters that were presented in Figure 1 were re-calculated based on normalization to interaction frequencies measured in a gene desert region on chromosome 3 [36] instead of to interaction frequencies measured at the TFIIH locus. Results indicate the average of three independent experiments +/− standard deviation. Values for the timepoint of maximum interaction frequency (6 h) were compared to values at time 0 by one-tailed t-test. ***p<0.001.
(EPS)
